# Augmented Reality: A Brand New Challenge for the Assessment and Treatment of Psychological Disorders

**DOI:** 10.1155/2015/862942

**Published:** 2015-08-03

**Authors:** Irene Alice Chicchi Giglioli, Federica Pallavicini, Elisa Pedroli, Silvia Serino, Giuseppe Riva

**Affiliations:** ^1^Applied Technology for Neuro-Psychology Lab, IRCCS Istituto Auxologico Italiano, 20145 Milan, Italy; ^2^Department of Psychology, Catholic University of Milan, 20123 Milan, Italy

## Abstract

Augmented Reality is a new technological system that allows introducing virtual contents in the real world in order to run in the same representation and, in real time, enhancing the user's sensory perception of reality. From another point of view, Augmented Reality can be defined as a set of techniques and tools that add information to the physical reality. To date, Augmented Reality has been used in many fields, such as medicine, entertainment, maintenance, architecture, education, and cognitive and motor rehabilitation but very few studies and applications of AR exist in clinical psychology. In the treatment of psychological disorders, Augmented Reality has given preliminary evidence to be a useful tool due to its adaptability to the patient needs and therapeutic purposes and interactivity. Another relevant factor is the quality of the user's experience in the Augmented Reality system determined from emotional engagement and sense of presence. This experience could increase the AR ecological validity in the treatment of psychological disorders. This paper reviews the recent studies on the use of Augmented Reality in the evaluation and treatment of psychological disorders, focusing on current uses of this technology and on the specific features that delineate Augmented Reality a new technique useful for psychology.

## 1. Introduction

Augmented Reality (AR) is a new technological system that allows inserting virtual contents in the real world in order to run in the same representation and, in real time, enhancing the user's sensory perception of reality [1]. Compared to a virtual reality system characterized by a computer-generated environment that elicits a strong user's experience of “presence” [2, 3], an AR system applies virtual and real elements in a real scene augmenting the user's perception of the world.

More precisely, Azuma et al. [4, 5] defined an AR platform as a system that [6]

- combines real and virtual objects in a real environment,
- runs interactively and in real time,
- registers real and virtual objects with each other.

**Figure 1:**
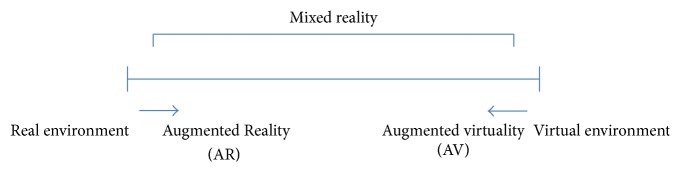
It shows a continuum between reality and virtual reality. Mixed reality is located between them and includes Augmented Reality (AR) and augmented virtuality (AV). AR is placed closer to real enviroment than virtual environment.

Furthermore, according to Milgram et al. [7, 8], AR places between reality (real environment) and virtuality (virtual environment) on the reality-virtuality continuum (see Figure 1).

In an AR system, users see an image made up of a real image and virtual elements that are superimposed over it. The addition of virtual elements may also inhibit the perception of real elements by overimposing the virtual elements on the real elements. Nevertheless, the most important aspect in AR is that the virtual elements provide the real world with remarkable and valuable information. The addiction of virtual elements may involve not only the view but also the hearing, smell, and touch [9].

From the point of view of technology devices, AR can be defined as a set of techniques and tools that allow adding information to the physical reality. Various technologies are used in AR rendering including handheld devices, display system worn on one's user (nonhandheld devices), and projection displays.

Modern handheld mobile computing like smartphones and tablets contain these elements, which include a camera and sensors such as accelerometer, Global Positioning System (GPS), and solid-state compass, making them a suitable AR platform. The smartphone or tablet take in real time the surrounding environment and the virtual elements are superimposed to the real world.

An example of nonhandled device is the head mounted display (HMD), a display system worn on one user's head such as a helmet or glasses. HMD systems are characterized by sensors that receive input information about the environment by the user's head movements adding at a later stage various virtual contents.

As regards the projection displays, the virtual elements are projected on the real objects in order to be augmented. The projection occurs with a single room-mounted projector without any display system worn on one user's head. The projector generates a virtual image on the room surface using an automated calibration procedure that takes into account the structure of the surface overlapping the virtual image.

Furthermore, an AR platform requires a software application able to augment the real world by using one or more hardware devices. Marker-based and markerless systems are the two main software applications used in AR system. The AR marker-based systems are stylized pictures in black and white that are recognized by the computer webcam and which are superimposed in real time multimedia contents: video, audio, 3D objects, and so forth. Instead, in the markerless [10] AR system the software application catches the user's positional and orientation data through GPS and compass device adding the virtual contents in an accurate position on or in the real environment.

To date, AR has been used in medicine [11], entertainment [12], maintenance [13], architecture [14], education [15, 16], and cognitive [17, 18] and motor rehabilitation [19–24] but very few applications of AR exist in clinical psychology and, in particular, it is still underused in the treatment of psychological disorders [25].

Starting from these premises, the aim of this paper is to review the recent studies on the use of AR in the evaluation and treatment of psychological disorders, focusing on current uses of AR in psychology and the various factors that make a new technique useful for the treatment of psychological disorders, expanding the possible fields of use of AR.

## 2. Materials and Methods

We followed the Preferred Reporting Items for Systematic Reviews and Meta-Analysis (PRISMA) guidelines [26].

### 2.1. Search Strategy

A computer-based search in several databases was performed for relevant publications describing the use of AR in psychology. Databases used for the search were PsycINFO, PubMed/Medline, and Web of Science (Web of Knowledge). We searched using the string “Augmented Reality” AND (psycholog^*^  OR assessment OR treatment). We excluded articles where the full text was not available or where the abstract lacked basic information for review. The first search was performed for publications in the English language, and then we decided to clean the results, considering only publications for the last ten years, from 2004 forward and eventually updated the search results through December 2014. Expert colleagues in the field were contacted for suggestion on further studies not considered in our search.

### 2.2. Selection Criteria

We have included articles on AR used for psychological settings in assessment or treatment studies. Excluded from the analysis were studies that omitted the inclusion criteria, non-English published studies, review articles, case reports, letters to the editor, research protocols, patents, and editorials. We tried to contact corresponding authors of the included studies with the intent of obtaining incomplete or supplementary data.

### 2.3. Quality Assessment and Data Abstraction

To assess a risk of bias, PRISMA recommendations for systematic literature analysis have been strictly followed. Three authors (Irene Alice Chicchi Giglioli, Federica Pallavicini, and Silvia Serino) independently selected paper abstracts and titles and analyzed the full papers that met the inclusion criteria, resolving disagreements through consensus.

The data extracted from each included study were sample type, study design, sample size, type of therapy or assessment, and selected findings.

## 3. Results and Discussion

### 3.1. Systematic Review Flow

**Figure 2:**
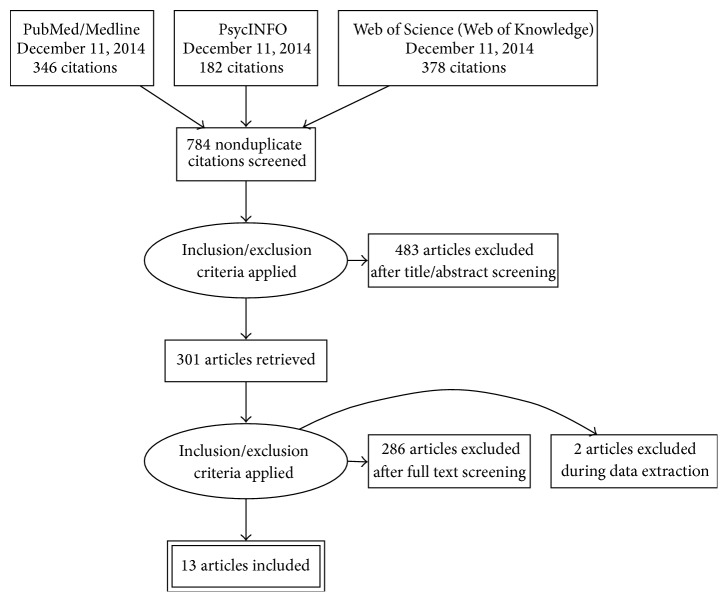
Flow diagram of study selection.

The flow chart of the systematic review is shown in Figure 2. Our initial search yielded 918 nonduplicate citations screened via PsycINFO, PubMed/Medline, Web of Science (Web of Knowledge), and other sources: more information is available in Search Strategy and supplement. After the application of inclusion/exclusion criteria, papers have been reduced to 301 articles. A more in-depth investigation of the full papers resulted in an exclusion of 286 articles. During the data extraction procedure, 2 additional full papers were excluded. In the end, 13 studies met full criteria and were included in this review (see Table 1).

### 3.2. Selected Studies on Augmented Reality

Despite a large volume of studies on AR, little has been done specifically to psychological assessment or treatment. In the current review we present a broad range of experimental and clinical studies.

### 3.3. Results

In the area of clinical psychology, a few but remarkable studies have met the DSM-IV-TR criteria [27], showing the usefulness of AR in the treatment of a specific phobia. More specifically, the review of the literature showed that the phobia of small animals (cockroaches and spiders) and the acrophobia [28–38] are the current areas that used AR in the assessment and treatment of specific psychological disorders. For the readability of the contents, we have divided the results section in two paragraphs based on specific phobia's typology. The studies are presented in chronological order for showing the developments and advancements that occurred in the use of AR in this area. In addition, the selected final studies can be found in Tables 2 and 3. Each of them is described below.

### 3.4. AR and Specific Phobia for Small Animals

In the studies, the AR exposure therapy was based on Öst et al.'s “one-session treatment” guidelines [41, 42]. Individuals make one single intensive exposure session that lasts up to three hours.

The first analysed study that used an AR system to assess and treat specific phobias was conducted by Juan et al. (2004) [39]. A single individual with cockroach phobia [27] was assessed using an HMD-AR system. The AR device used was an HMD system connected with a camera and a PC. The camera, placed on the HMD, recognized the marker through the movement of the subject's head, projecting the virtual cockroaches in front of the subject. The AR single exposure session consisted progressively in seeing, touching, and finally killing one or more virtual cockroaches. The therapist chose in any moment how many cockroaches had to appear on the scene, their size, and if they had to move or not. During the treatment, the augmented cockroaches were able to arouse anxiety in patient that decreased after an hour of exposure. More specifically, before, during, and after treatment, the patient ranked her level of anxiety on a 10-point scale (where 0 represents no anxiety and 10 very high anxiety) using the Subjective Units of Discomfort Scale (SUDS) [44]. The data showed a decrease in anxiety score after exposure (with a score of 10 on SUDS at the beginning of the session and a score of 0 after the session) and clinical improvements regarding patient's phobia. In particular, after exposure, the patient was able to approach, interact, and kill real cockroaches.

Botella et al. (2005) [35] assessed a cockroach phobia case study [27] using an HMD-AR system developed for this specific disorder. The AR device used was an HMD system connected with a camera and a PC. The camera, placed on the HMD, recognized the marker through the movement of the subject's head, projecting the virtual cockroaches in front of the subject. The AR system included the possibility for the therapist to choose the number of cockroaches, their size, and movements and for the patient to kill one or more cockroaches using two different instruments, a fly swatter or a cockroach killer. Depending on the selected tool the system played a sound analogous to the real one. In order to assess the intensity of the phobia, the behaviour avoidance test (BAT) [42], degree of belief in catastrophic thought (assessed daily on scale from 0% to 100%), Fear and Avoidance Scales [45],* *Fear of Spiders Questionnaire (FSQ) [46], Spider Phobia Beliefs Questionnaire (SPBQ) [47], and Subjective Units of Discomfort Scale (SUDS) [44] were used. Furthermore, in order to assess the degree of presence and reality judgment experienced in the AR session, the authors created specifically for this study one ad hoc questionnaire composed by two questions related to presence: “To what degree have you felt present in the situation?” and “To what degree have you felt that you were in the place where the cockroaches appeared?” and one question for the reality judgment: “To what degree did the cockroaches appear to be real?” After AR exposure, the participant was asked to evaluate these features on a scale from 0 (no degree of being in a place/being real) to 10 (very high degree of being in a place/being real). The results showed that, before the exposure, patient exhibited a considerable fear and avoidance behaviours and after exposure not only were there important decreases in the fear and avoidance scores, but also the patient was able to approach, interact, and kill cockroaches with a high degree of presence and reality judgment. Similarly, at the beginning of the experiment, virtual cockroach induced anxiety in patient but after one hour of exposure the anxiety was significantly declined. Finally, the treatment gains were maintained in a follow-up conducted two months after the end of the treatment, showing decreases in the various scales of the BAT [42].

Juan et al. (2005) [31], for the first time, evaluated the effectiveness of an AR system not in one single case but in a sample of nine patients with cockroach and spider phobia [27]. The AR system was the same used in the Botella et al. study [35], described previously. The AR exposure involved the gradual appearance of one or more spiders/cockroaches and the possibility for the patients to approach them with hands, to look in boxes in order to simulate when you are searching for a small animal in your house, and to beat and throw away them. With respect to psychological measures, anxiety, fear, and avoidance behaviours were assessed using Fear and Avoidance Scales [45] and SUDS [44]. The degree of presence and reality judgment experienced by the users in the AR system were assessed using three ad hoc questions, created specifically for this study. The three questions were “To what degree have you felt present in the situation?” “To what degree have you felt that you were in a place in which spiders/cockroaches appeared?” And “To what degree did you think the spiders/cockroaches were real?” Participants were asked to evaluate these features in a scale from 0 to 10 (where 0 represents the lowest degree of being in a place/being real and 10 the highest degree of being in a place/being real). The results showed that the treatment produced a decrease in the patient's fear and avoidance behaviours [45] when they had to face with the target spider/cockroach. Furthermore, as in the study of Botella et al. [35], during the exposure, the participant's anxiety scores (SUDS) [44] were high, but they diminished at the end of the treatment.

In Botella et al. (2010) [34] an AR system was tested in the short and long term (three-, six-, and twelve-month follow-up) on a sample of six individuals suffering from cockroaches phobia [27]. The AR system was the same as that used in the preceding studies [31, 35]. Before, during, and after the AR exposure, participants were asked to fill out questionnaires, including SUDS [44] to evaluate anxiety levels, BAT [42], FSQ [46], and SPBQ [47] to assess fear and avoidance behaviours. AR exposure has been led in a single extended session lasting up to three hours. Each participant faced with various scenarios, progressing from the easiest to the most difficult situation. For example, at the beginning of the exposure, the program exhibited one cockroach to the participants, and more animals were added progressively. The purpose of the exposure was to interact with many cockroaches repeatedly, touch them, kill them, and remain in the situations until they experienced a considerable decrease in anxiety. As above in Botella et al. (2005), the results showed that AR was effective in treating cockroach phobia, improving significantly in all outcome measures after treatment. More specifically, the AR system, at the beginning of the exposure, was able to induce anxiety in the participants and after treatment produced a significant decrease in the level of fear and avoidance behaviours in all participants. In accordance with the BAT's scores [42], before treatment, none of the participants were able to interact with a real cockroach, while after treatment all participants could approach it. At the same time, also the self-report scores of FSQ [46] and SPBQ [47] improved significantly after treatment. Furthermore, the treatment gains were maintained at three-, six-, and twelve-month follow-up periods. Unlike the study conducted in 2005 by Botella et al. [35], measures related experience has not been recorded.

Bretón-López et al. (2010) [32] evaluated the ability of an AR system to raise anxiety and, secondly, to elicit sense of presence and reality judgment in six participants suffering from cockroach phobia [27]. In the single AR exposure session, participants were exposed to various stimuli, based on each individual's hierarchy of fears (from one static insect to the one in movement, from more static insects to ones in movement, and from insects next to personal belongings to those next to hands). Moreover, the AR system included the possibility to vary the numbers of cockroaches on the scene and the movement and the size of cockroaches. During and after the AR exposure, patients' level of anxiety was evaluated through the SUDS on a scale of 0 (no anxiety) to 10 (very high anxiety) [44], and the degree of presence through the Presence and Reality Judgment Questionnaire (PRJQ) [48]. The data showed that, at the start of AR immersion, the anxiety levels, measured through the SUDS questionnaire [44], ranged from 9 to 10 (the highest levels of the scale) but they decreased progressively during the exposure. The levels and the duration of exposure needed for anxiety reduction were based on initial levels of fear and on the severity of the phobia. Furthermore, patients showed high scores in the PRJQ [48], representing high levels of presence and reality judgment experienced during the AR exposure.

In 2011, Botella et al. [33] assessed a single cockroach phobia case study [27] testing an AR system using a mobile phone and creating a mobile game, “Cockroach Game” [33] for the treatment of this phobia. The subject conducted three therapeutic phases. In the first phase, the participant was asked to play, as much as she wanted, “Cokroach Game” for nine days and to record her levels of fears, avoidance, and belief in the catastrophic thought using the BAT [42] and FSQ [46]. In the second phase, the participant received the AR exposure treatment, assessing again the psychological measures. In the third phase, the participant was asked to play again the “Cockroach Game” for another period of nine days and recorded the same questionnaire. In this study the experiential measures of presence and reality judgment have not been taken into account. The data showed that before and after the first phase a slight improvement is obtained in performance, fear, and avoidance, whereas there was an increased in the belief in the catastrophic thought. After the AR exposure there were improvements in all BAT measures [42]: performance, fear, avoidance, and belief. After the third phase a significant decrease was obtained in all psychological measures, maintaining them at one, three-, six-, and twelve-month follow-up periods.

In 2011, Juan and Calatrava [37] compared an AR optical see-through (OST) system with a video see-through (VST) for the treatment of spiders and cockroaches phobia in twenty-four nonphobic participants. Individuals were divided in low and high fear subject's group according to scores in the fear and avoidance of cockroach and spider questionnaires [46] and underwent both experimental conditions. Before, during, and after exposure subjects were asked to rate their level of anxiety from 0 (no anxiety) to 10 (very high anxiety). Presence experienced by participants was assessed using an adapted version of the Slater et al.'s (1994) questionnaire [49]. The six question were “Please rate your sense of being in a room where there are cockroaches/spiders.” “To what extent were there times during the experiment when the cockroaches/spiders were real for you?” “When you think back to your experience, do you think of cockroaches/spiders more as images that you saw (a movie, a picture), or more as cockroaches/spiders that were in the same room as you were?” “During the experiment which was strongest on the whole: your sense of being in the room where there were cockroaches/spiders, or your sense of being in a room without cockroaches/spiders?” “Think about your memory of being in a room where there were cockroaches/spiders. How similar is this memory to your memories of other places where there were these animals?” And “During the experiment, did you often think that you were actually in a room where there were cockroaches/spiders?” The scoring was on a scale of 1–7 (where 0 corresponds to not being in a place and 7 represents the normal experience of being in a place). Results showed that the VST system induced a higher sense of presence than the OST system and the two systems produced similar and significant anxiety before treatment that decreased after exposure.

Juan and Joele (2011) [38] compared an AR visible marker-based system with an AR invisible marker system in twenty-four healthy subjects. Participants rated their intensity of anxiety level from 0 (no anxiety) to 10 (very high anxiety) at eight different moments during the AR exposure. As in the previous study, after each AR exposure, individuals were asked to fill out an adapted Slater et al.'s (1994) questionnaire (SUS) [49] to rate the subjective feelings of presence experienced. Results showed that the AR invisible marker system elicited a higher sense of presence compared to the AR visible marker system. Furthermore, at the beginning of the treatment, the AR invisible marker system provokes a higher level of anxiety that decreases significantly during and at the end of the AR exposure.

Wrzesien et al. (2001a; 2011b) [28, 29] conducted two studies to evaluate the level of anxiety, avoidance, behavioural avoidance, and belief in negative thoughts in patients with small animal phobia [27].

The first study [29] compared the in vivo exposure therapy with the AR exposure therapy in twenty-two individuals with specific phobia for spiders and cockroaches [27]. The patients were randomly allocated to one of two groups. Before and after the exposure session, the participants were asked to fill out the behaviour avoidance test (BAT) [42]. The data showed that both in vivo and AR exposure are therapeutically effective in reducing anxiety, avoidance, and behavioural avoidance. In particular, the analysis of the pre- and posttest BAT scores showed no statistically significant differences between the in vivo group and the AR exposure group. Furthermore, an intragroup analysis showed a statistically significant decline in the severity of avoidance under both conditions, suggesting that both exposures are effective in the reduction of avoidance behaviours after treatment.

In the second study [28], five patients were assessed and treated using only an AR therapeutic exposure [27]. Before, during, and after the exposure session, the participants were asked to rank their level of anxiety on a scale of 0 (no anxiety) to 10 (very high anxiety), avoidance on a scale of 0 (low degree of avoidance) to 10 (high degree of avoidance), behavioural avoidance on a scale of 0 (low degree of behavioural avoidance) to 13 (very high degree of behavioural avoidance), and belief in catastrophic thoughts on a scale of 0 (low degree of belief) to 10 (high degree of belief). The results showed a posttreatment decrease in level of anxiety, avoidance, and belief in negative thoughts. More precisely, if, before the therapy, patients were unable to get close to live cockroaches, after the treatment they were able to interact with real cockroaches into a terrarium.

In a further study, Wrzesien et al. (2013) [30] experimented with an innovative technological AR system named therapeutic lamp (TL). The TL is an AR display projector created for the treatment of small animals' phobia. The trial included twenty-six healthy volunteers and consisted of one single exposure session composed by twelve exercises progressed from those who induced less anxiety to the ones that elicited more anxiety. For example, at the beginning of the exposure, participants observed three dead and three paralyzed animals and, at the end, they had to kill 30 animals with the flyswatter. In order to measure the participants' experience during the exposure, four clinical instruments were used. The Spider and Cockroach Anxiety and Avoidance Questionnaire was assessed before the session on a scale of 0 (no degree of fear and avoidance) to 7 (high degree of fear and avoidance). The Self-Efficacy Belief Questionnaire was used before and after the exposure session on a scale of 0 (no degree of belief that the participant could confront a real cockroach or spider) to 7 (high degree of belief). The Subjective Units of Discomfort Scale (SUDS) [44] was assessed at the beginning and end of each exercise in the session on a scale to 0 (no anxiety) to 10 (very high anxiety). The Presence and Reality Judgment Questionnaire (PRJQ) [48] was tested at each exercise's start on a scale of 0 (no degree of being in a place/being real) to 10 (high degree of being in a place/being real). The data showed that the participants' anxiety scores, measured by SUDS [44], were high at each exercise's beginning but decreased after the exercise session. In addition, the participant's belief in their capacity to face with the small animals improved significantly after the session. Finally, the PRJQ [48] scores showed that the participants felt the virtual animals' presence relatively well and considered them to be rather real. Therefore, the authors concluded that the AR-TL could be a good and helpful treatment's tool for psychological disorders even if the system has to be validated with patients in future studies.

All these studies represent new potentiality and possibility of assessment and treatment in the area of psychological disorders. However, they disclose some limitations. The majority of the disclosed studies [28, 29, 31–35] include a too small sample for the experimental validity characterized by patients with specific phobias. Instead, one study [30] focused on testing an innovative AR system with healthy volunteers in order to verify the efficacy, usability, and quality of user's experience of the new platform. Related to the previous consideration, the presented studies [28, 30–35] have not included control groups, experimental controls, or randomized controlled studies. Only one study conducted by Wrzesien et al. (2011a) [29] has performed a randomized controlled study, comparing the in vivo exposure therapy with the AR exposure therapy.

### 3.5. AR and Acrophobia

In 2006, Juan et al. [43] advanced the use of immersive photography in an AR system to treat acrophobia. For evaluating this system, forty-one healthy volunteers walked around at the top of a staircase in a real environment and using the immersive photography environment. After their experience, the participants filled out the SUS questionnaire [49] to assess their subjective sense of presence. The data showed that the AR condition induced a sense of presence equal to the one experienced by the subjects in the real world.

Another study, conducted by Juan and Prez (2010) [36], compared an acrophobic virtual reality (VR) and AR environment assessing differences in the sense of presence and anxiety elicited by the two systems. Twenty healthy participants underwent both experimental conditions and after using each system (AR or VR), they completed an adapted SUS questionnaire [49]. Moreover, at six different moments during the two experiences, the participants were also asked to rate their anxiety level from 0 (no anxiety) to 10 (very high anxiety). Regarding the sense of presence, the results showed no differences between the two systems. Moreover, data revealed that anxiety levels decrease after the exposure.

### 3.6. Discussion

In the current scientific scenario, AR is a relevant issue and offers a viable alternative to traditional methods as the in vivo exposure therapy. This paper is the first systematic review that explores studies in the literature that use AR as a tool in the treatment of psychological disorders.

To date, the analysis of the literature suggested that AR has been mainly used in the evaluation and treatment of specific phobias such as phobias for small animals (cockroaches and spiders) and acrophobia. Regarding the assessment and treatment of specific phobias, from the literature analysis, it has been observed that thirteen studies have used an AR system in order to reduce anxiety, fear, and avoidance behaviours. Specifically, eleven studies concerned the evaluation and treatment of cockroaches and spiders phobia, while two studies affected the acrophobia.

As regards the cockroaches and spiders phobia, nine studies applied an AR-HMD system connected with a camera and a computer [28, 29, 31, 32, 34, 35, 37–40]. The camera was placed on the HMD helmet and connected with a USB to the computer where the AR system ran. The camera, recognizing the marker through the movement of the subject's head, projected the virtual images and objects in front of the subject. Among these studies, two [37, 38] compared different AR systems in order to evaluate their efficacy. In particular, Juan and Calatrava (2011) [37] compared an AR optical see-through (OST) system with a video see-through (VST), and Juan and Joele (2011) [38] contrasted an AR visible marker-based system with an AR invisible marker system.

The last studies have tested two innovative technological AR systems [30, 33]. In 2011, Botella et al. [33] have experimented with a new AR-mobile phone system and have created a mobile game, “Cockroach Game” [33] for the treatment of the cockroach phobia. The Cockroach Game is a puzzle game characterized by different levels of fear stimuli divided in two situations allowing players to progress in the game. The first included the possibility to see animals on various virtual situations inside the mobile phone. The second one allowed seeing them on real environment such as on the hands. In a further study, Wrzesien et al. [30] experiment with an innovative technological AR system, named therapeutic lamp (TL). The TL is an AR display projector created for the treatment of small animals' phobia.

As mentioned above, the majority of the studies concerned the assessment and the treatment of small animals phobias: five studies have focused on patients with cockroaches' phobia [32–35, 39, 40] and three on the cockroaches and spiders phobia [28, 29, 31] according the DSM-IV [27] criteria. Instead, three studies [30, 37, 38] have included healthy volunteers without any diagnosis of psychological and medical problems. In most of the studies [28, 29, 31–35], the AR exposure therapy was applied using the “one-session treatment” guidelines developed by Öst [41, 42].

A remarkable feature refers to the number of subjects included in these studies and the psychological and experiential measures in the assessment of the AR exposure therapy. Three studies experimented with one case study [33, 35, 39], two studies tested the AR system on six patients [32, 34], and one study assessed nine patients [31]. The remaining five studies have experimented with a higher number of subjects [28–30, 37, 38]. In 2011, Juan et al. [37, 38] assessed twenty-four healthy volunteers, while Wrzesien et al. (2011a; 2011b; 2013) [28–30], in the first two studies [28, 29], assessed twenty-two patients and, in the third study [30], they tested twenty-six healthy subjects.

Furthermore, all these studies considered anxiety as psychological measure showing that AR elicits anxiety as soon as the stimulus appeared that decreased during the time of exposure [28–35, 37–39]. Among these studies, seven of them also considered fear and avoidance behaviours showing that AR was able to reduce significantly fear and avoidance behaviours after the stimuli exposure [28–31, 33–35]. Finally, as regards the quality of user's experience, five studies measured presence [31, 32, 35, 37, 38] and three of them evaluated reality judgment [31, 32, 35] showing a high sense of presence in the AR system and reality judgment of the small animals.

Taking the two acrophobia studies [36, 43] into account, both applied an AR-HMD system to evaluate its efficacy comparing, in the first study, the AR-HMD system with a real environment [43] and, in the second study, the AR-HMD system with a VR- HMD system [36].

Regarding the number of subjects, both of the studies included healthy volunteers and, specifically, in 2006, Juan et al. [43] assessed forty-one participants, while in 2010 they evaluated twenty [36].

Among these studies, just Juan and Prez (2010) [36] considered anxiety as psychological measure showing that AR is effective as VR to induce anxiety when the stimulus appeared, which decreased during the exposure. In conclusion, both studies assessed the sense of presence showing, in the study of 2006 [43], a higher sense of presence in the AR environment than the real environment and in the study of 2010 [36] no difference between the AR and the VR environment.

The studies mentioned above suggest that AR offers many advantages such as the possibility to reproduce real objects in the environment (ecological validity), controlled situations, and ad hoc environments and objects in order to tailor the situations on the subject's needs and therapeutic purposes. However, the discussed studies are mostly preliminary researches that disclose some limitations due to the fact that AR exposure has only been recently tested for the evaluation and treatment of psychological disorders. The majority of the disclosed studies [28, 29, 31–35, 39] include a too small individual's sample and only one [29] of them is based on a randomized controlled design.

Instead, the reaming studies focused on testing the AR system on healthy subjects in order to verify the efficacy, usability, and the quality of user's experience of the new platform using randomized controlled studies [30, 36–38, 43].

Apart from these studies, the literature is lacking studies of AR on the treatments of other several psychological disorders.

Currently, other technological devices have proven effective in the evaluation and treatment of psychological disorders. In particular, VR has been demonstrated to be a very useful tool for the treatment of several psychological problems such as eating disorders and anxiety disorders [50–54]. The traditional treatment for these psychological disorders is the in vivo exposure therapy. As the term implies, the in vivo exposure therapy allows subjects to experience their fears under the close supervision of a physician or a therapist. However, not all patients benefit from this treatment and, according to Jefferey et al. (2000) [55] and Mann et al. (2007) [56], some patients do not improve after treatment and others relapse in the long term.

In the last few years many studies have showed the efficacy of VR environments as therapeutic tool [50–53, 57–60]. A VR environment is a completely simulated three-dimensional environment modelled by a computer that allows, through the simulation, coping with critical and fear situation in a safe condition without losing sensory experience and physical presence.

A feature that makes VR a useful tool for evaluation and treatment of psychological disorders is to elicit emotional responses commensurate with the real ones.

The emotional engagement in virtual exposure depends on a number of factors such as the sense of presence. The sense of presence in virtual exposure is defined as the degree of “being there” in the virtual environment [61] and is marked by the sense of immersion and a sense of interaction. The sense of immersion is the result of the technological tools used such as the use of the HMD device that allows an immersive 3D experience to subjects. Instead, the sense of interaction is defined as the degree of interaction and manipulation for individuals of the virtual content or environment. Therefore, a high sense of presence in a virtual environment provides a greater realistic perception of the experience and consequently a strong and deep emotional engagement. This experience increases the ecological validity of the instrument of VR in the treatment of psychological disorders ensuring, through a simulated environment, a similar experience to the real one. At the same time, the user, feeling “present” in the simulated environment, perceives it as real and tends to transfer the expected skills from the virtual world to the real one in a nearly automatic manner.

Recent studies have shown that virtual exposure is as effective as in vivo exposure [62–64] and, in particular, the exposure to virtual environments has produced emotional and behavioural responses similar to those that occurred in the real world.

In the studies of Ferrer-García and Gutiérrez-Maldonado [62, 63] six virtual environments emotionally relevant and significant to subjects with eating disorders (ED) have been realized. Before and after exposure to each virtual environment, they estimated the state of anxiety and depression levels and the data showed that the virtual exposure is effective in causing and provoking relevant emotional responses to subjects.

Gorini et al. [64] started from the two previous studies but compared the virtual stimuli with the real ones and with their correspondent pictures to test the psychological (measuring the level of anxiety and sense of presence) and physiological (measuring the heart and respiration rate and the skin conductance) reactions to food in a sample of ED patients and healthy controls. After each experimental condition, in order to estimate the psychological variations, the subjects carried out two states of anxiety tests (STAI-S and VAS-A) and then the virtual exposure completed the presence questionnaire. The data showed that virtual stimuli are effective as the real one and more than photographs in eliciting emotional responses in ED patients and, more generally, the use of VR instead of real stimuli may simplify the framework of very specific contexts to help patients to cope with their conditions through a very controlled stimulation.

Finally, regarding the sense of presence, the results showed a significant degree of presence on the level of state anxiety in VR and real exposure conditions. As mentioned above, the VR gives the possibility of the subject to manipulate and interact with the environment and contents as in the real world.

Similarly, AR could be considered a useful tool in the evaluation and treatment of psychological disorders providing a number of advantages as the VR. Indeed, the AR, as VR simulation, can be seen as an experiential process, and the experience is an essential component in dealing with critical situations. Furthermore, this experience could allow exploring environments and situations hard to reproduce in reality and, as the VR, the feeling of presence in the AR system could permit the user to assign the learned behaviours from the AR world to the real one.

The AR simulation, therefore, could be considered an efficient method to act on real behaviours avoiding the risks and complications typical of real environments.

## 4. Conclusions

The aim of this paper was to review the recent studies on the use of AR in the evaluation and treatment of psychological disorders, focusing on current uses of AR in psychology and the various factors that make a new technique useful for the treatment of psychological disorders, expanding the possible fields of use of AR.

In general, the presented studies show that the AR seems to be a promising and useful tool for intervention in the treatment of specific phobias. Nevertheless, the small sample of subjects examined and the lack of control group and randomized controlled studies necessitate more randomized controlled experiments for exploring the AR efficacy in the clinical treatments. Despite these limitations, AR is proving to be a new technique useful to patients to experiment with technologically different and severe situations, as the exposure to fear or phobic stimuli, in a safe environment under the control of the therapist. Indeed, an AR system extends interactivity for assessing and supervising patient's reactions in real time and adaptability for creating controlled exposure settings based on the patient's needs or therapeutic purposes. Furthermore, it is to be noted that AR allows subjects/patients to manipulate and control the virtual elements, interacting with virtual objects placed in the real world in real time.

As a consequence, the experience to amplify the physical world with virtual contents can improve the ecological validity of the “mixed reality” [7] on environment, augmenting the sense of presence and engagement of the subject/patient. Indeed, studies of VR have shown that virtual stimuli are comparable to the real stimuli with regard to emotional responses [62–64]. Finally a strong and deep sense of presence and engagement can, also, improve the adherence to treatment.

Overall, AR may represent a new challenge for the assessment and treatment of different kinds of psychological disorders, such as eating and anxiety disorders performing new studies based on systematic measures of psychological and neurophysiological effects.

## Figures and Tables

**Table 1:** Detailed search strategy.

	“Augmented Reality” and psycholog^*^	Assessment	Treatment	Other sources	Total
Medline	23	56	267		346
PsycINFO	133	21	28		182
Web of Science (Web of Knowledge)	69	145	164		378

**Total**	225	222	459	12	**918**
**Nonduplicated**	191	203	378	12	**784**
Excluded (after reading title and abstract)					**483**
Retrieved					**301**
Excluded (after applying inclusion criteria)					**286**
Excluded (missing experimental data)					**2**
Included					**13**

^*^is a Jolly characters that means that the search strategy included terms as psychology and/or psychological.

**Table 2:** Information about the selected studies on the assessment and treatment of specific phobia for small animals using an AR system.

Authors	Year	Sample	Conditions	Dependent variables	AR device	Results
Juan et al. [39]	2004	1 patient with cockroach phobia	Case study with AR	Anxiety	HMD with AR-tags	Decrease of anxiety level after treatment

Botella et al. [35, 40]	2005	1 patient with cockroach phobia	Case study with AR	Avoidance behaviour Degree of belief in catastrophic thought Anxiety Target behaviour Presence Reality judgment	HMD with AR-tags	Decrease of anxiety, fear, and avoidance after treatment High level of presence and judgment of reality

Juan et al. [31]	2005	9 patients with cockroach and spider phobia	AR	Anxiety Fear Avoidance behaviours Presence Reality judgment	HMD with AR-tags	Decrease of anxiety, fear, and avoidance after treatment High level of presence and judgment of reality

Botella et al. [34]	2010	6 patients with cockroach phobia	AR	Anxiety Target behaviour Behavioural avoidance	HMD with AR-tags	Decrease of anxiety, fear, and avoidance after treatment and maintained at follow-up periods (three, six, and twelve months follow-up)

Bretón-López et al. [32]	2010	6 patients with cockroach phobia	AR	Anxiety Presence Reality judgment	HMD with AR-tags	Decrease of anxiety. High levels of presence and reality judgment

Botella et al. [33]	2011	1 patient with cockroach phobia	Case Study with AR	Anxiety Target behaviours Behavioural avoidance	Mobile phone marker less versus HMD AR-tags	Decrease of anxiety, fear, and avoidance behaviours in both conditions.

Juan and Calatrava [37]	2011	24 healthy volunteers	AR-OST HMD AR-VST	Anxiety Presence	AR-OST HMD versus AR-VST	The AR-VST induced grater sense of presence than AR-OST. Significant anxiety in both conditions

Juand and Joele [38]	2011	24 healthy volunteers	AR marker-based versus invisible AR-tags	Anxiety Presence	HMD with visible AR-tags versus invisible AR-tags	The invisible AR-tags induced a higher sense of presence and anxiety than the visible AR-tags

Wrzesien et al. [29]	2011a	22 patients with cockroach and spider phobia	In vivo versus AR	Anxiety, behavioural avoidance Belief in negative thoughts	HMD with AR-tags	Decrease of anxiety, fear, avoidance behaviours, and belief in negative thoughts

Wrzesien et al. [28]	2011b	22 patients with cockroach and spider phobia	AR	Anxiety, behavioural avoidance Belief in negative thoughts	HMD with AR-tags	Decrease of anxiety, fear, avoidance behaviours, and belief in negative thoughts

Wrzesien et al. [30]	2013	26 healthy volunteers	AR	Anxiety Avoidance	Therapeutic lamp (TL)	Decrease of anxiety

**Table 3:** Information about the selected studies on the assessment of acrophobia using an AR system.

Authors	Year	Sample	Conditions	Dependent variables	AR device	Results
Juan et al. [43]	2006	41 healthy volunteers	Real environment versus AR environment	Sense of presence	AR-HMD	High sense of presence in the AR environment

Juan and Prez [36]	2010	20 healthy volunteers	AR system versus VR system	Sense of presence Anxiety	HMD-AR with tags VR-HMD	In regard to sense of presence and anxiety levels, AR is effective as VR

## Abbreviations

AR: Augmented Reality
BAT: Behaviour avoidance test
FSQ: Fear of Spiders Questionnaire
GPS: Global Positioning System
HMD: Head mounted display
OST: Optical see-through
PRISMA: Preferred Reporting Items for Systematic Reviews and Meta-Analysis
PRJQ: Presence and Reality Judgment Questionnaire
SPBQ: Spider Phobia Beliefs Questionnaire
SUDS: Subjective Units of Discomfort Scale
TL: Therapeutic lamp
VR: Virtual reality
VST: Video see-through.
